# One method to establish Epstein‐Barr virus‐associated NK/T cell lymphoma mouse models

**DOI:** 10.1111/jcmm.14057

**Published:** 2018-11-28

**Authors:** Weili Xue, Weiming Li, Yufeng Shang, Yanjie Zhang, Xuan Lan, Guannan Wang, Zhaoming Li, Xudong Zhang, Yue Song, Baopeng Wu, Meng Dong, Xinhua Wang, Mingzhi Zhang

**Affiliations:** ^1^ Department of Oncology The First Affiliated Hospital of Zhengzhou University Zhengzhou China; ^2^ Henan Jonint International Research Laboratory of Lymphoma The First Affiliated Hospital of Zhengzhou University Zhengzhou China; ^3^ Henan University of Traditional Chinese Medicine Zhengzhou China; ^4^ Department of Pathology The First Affiliated Hospital of Zhengzhou University Zhengzhou China; ^5^ Lymphoma Diagnosis and Treatment Center of Henan Province Zhengzhou China; ^6^ The Boiler & Pressure Vessel Safety Inspection Institute of Henan Province Zhengzhou China

**Keywords:** methods to establish mouse models, mouse models, NK/T cell lymphoma

## Abstract

Novel nude mice model of human NK/T cell lymphoma were established by subcutaneously injecting two NK/T cell lymphoma cell lines into the right axillary region of mice and successful passages were completed by injecting cell suspension which was obtained through a 70‐μm cell strainer. These mice models and corresponding cell clones have been successfully developed for more than 8 generations. The survival rates of both resuscitation and transplantation in NKYS and YT models were 90% and 70% correspondingly. Pathologically, the tumour cells in all passages of the lymphoma‐bearing mice and cell lines obtained from tumours were parallel to initial cell lines. Immunologically, the tumour cells expressed the characteristics of the primary and essential NK/T lymphomas. The novel mice models maintained the essential features of human NK/T cell lymphoma, and they would be ideal tools in vivo for further research of human NK/T cell lymphoma.

## 1 INTRODUCTION

Extranodal natural killer (NK)/T cell lymphoma (ENKTCL) is an aggressive malignancy of putative NK cell origin, with a minority deriving from the T cell lineage. Pathologically, the malignancy occurs in two forms, extranodal NK/T cell lymphoma, nasal type (NNKTCL); and aggressive NK cell leukaemia.^1^ Although chemotherapy and radiotherapy can improve the disease outcome, the prognosis of NKTCL is poor, and no targeted therapy is currently available.^2^, ^3^, ^4^, ^5^, ^6^ The pathogenic mechanisms of NK/T cell lymphoma remain largely unclear, and further investigation may aid in improving the accuracy of lymphoma diagnosis and gene therapy.^7^ Meanwhile, lymphoma is one immune‐related disease,^8^ the microenvironment in vivo matters a lot in the mechanism of lymphoma. Complex biological processes often require in vivo analysis, and many important research advances have been made using mice as a model for the study of various biological systems. Mouse models are irreplaceable tools for the study of carcinogenesis. The mouse shares anatomical, immunological, and genomic similarities with humans and is the most accessible model system.^9^ To clarify the molecular pathogenesis of NKTCL, we performed establishment of mouse models in NKTCL, because animal models enable elucidation of the pathological and physiological mechanisms underlying lymphomas and preclinical testing of new drugs. Meanwhile, lymphoma mouse models with stable passages have various advantages over cell lines in vitro and single mouse models that have no passage in lymphoma research in vivo. It is not only an efficient in vivo platform to investigate drug efficacy and resistance studies in various cancers, but likely to be applied to lymphoma therapy. There is a growing need for mouse models that have stable characteristics to mimic in vivo studies of NK/T cell lymphomas. Here we introduce two NK/T cell lymphoma mouse models we have established successfully.

## 2 MATERIALS AND METHODS

### 2.1 Cell lines

The human NK/T cell lymphoma cell lines YT and NKYS were gifts of Dr. John Chen in City of Hope. The origin of cell lines has been confirmed by next‐generation sequencing. Every cell lines were transfected with luciferase to get stable cell lines. YT and YT‐luciferase were maintained in Roswell Park Memorial Institute 1640 (RPMI1640, Gibco, Beijing, China) supplemented with 10% foetal bovine serum (FBS; HyClone Laboratories, Logan, UT, USA), NKYS and NKYS‐ luciferase were maintained in RPMI1640 supplemented with 10% FBS and 100 IU/mL rhIL‐2. All cells were incubated at 37°C in a humidified atmosphere containing 5% CO_2_.

### 2.2 Nude mice model establishing

Three‐ to four‐week‐old female BALB/c (nu/nu) nude mice and NOD/SCID mice were bought from Beijing Vital River Laboratory Animal Technology Co., Ltd. (Beijing, China), weighed approximately 15–20 g, fed in the specific pathogen‐free (SPF) barrier system and provided sterile food and water. All procedures were performed under aseptic condition. Before animal experiment, mice were fed adaptively for 1 week in the new circumstances and cyclophosphamide (CTX) for 2 days before cell suspension injection and transplantation. Cells were washed repeatedly in Hank's liquid, resuspended in 0.2 mL RPMI1640 culture media and subcutaneously injected into the right axillary region of mice (NKYS cells in NOD/SCID mice and YT cells in nude mice). The NKYS models were injected with 20 000 IU/kg rhIL‐2 once a week intraperitoneally. The general condition and the appearance changes of the lymphoma bearing mice were observed every day, and the size of tumour was measured in length and width three times a week. As soon as the tumour reached about 2000 mm^3^ in size or 1.0‐2.0 cm at length, or the animal showed distress, the mouse was killed. A part of the tumour tissues was used for continuous transplantation, the remaining part was divided into two parts, one for routine morphologic observation and the other preserved in liquid nitrogen for further use.

### 2.3 Bioluminescent imaging

To investigate whether the tumour existed in other part of mice, tumour development was quantified by in vivo imaging using an IVIS Lumina III system (PerkinElmer, Waltham, MA, USA). Mice were anaesthetized with isoflurane, injected i.p. with 150 mg/kg body weight Luciferin (PerkinElmer) and imaged 5 minutes after Luciferin injection. Total flux values, which correlate directly with tumour mass, were measured. Images were analysed with Living Image 3.0 software (PerkinElmer).

### 2.4 Passage

A period of time after cell suspension injection (at least 35 days in NKYS cells and 45 days in YT cells), the tumour‐bearing mice (denoted F0) were killed. We took the passage in three ways initially. Inserting the small block from the tumour into the next mice is the first. However, both the tumorigenecity and growth rate were quite low, so lastly we abandoned this method. A part of tissues excised from the tumour were minced and strained through a 70‐μm cell strainer (Becton Dickinson, San Jose, CA, USA). One part of the cells obtained through the strainer was injected in the same manner as for the establishment of first mice model.^10^ Others were cultured and began to proliferate. In the case of engraftment, 1.5 × 10^7^ to 5 × 10^7^ tumour cells were serially subcutaneously injected into the right axillary region of other mice. Stable mouse models were considered to be established based on the success of over six serial passages of the tumours (F5). Currently, the tissues and cells obtained from F0, F4, and F8 mice were used in the subsequent analyses. Notably, cells were injected with matrigel in the ratio of 1:1.

The cultured cells from every passage tumour have been always maintained. Cytocentrifuge smears of the cells were prepared and stained with May‐Giemsa for morphologic evaluation. To confirm that the tumour cells were clonally related to the original tumours, the sequences of the surface markers and EBV‐encoded RNA (EBER) in situ hybridization were analysed. All the animal experimental procedures complied with the Regulations regarding Animal Experiments in Zhengzhou University. The cell lines transfected with luciferase were passaged once or twice to observe the distribution of tumour cells in vivo.

### 2.5 Time course engraftment analyses after transplantation

To investigate the pattern of engraftment and proliferation of the lymphoma cells in the mouse models, time‐lapse engraftment analyses were performed. After subcutaneous injection of 2.0 × 10^7^ tumour cells in the right axillary region of mice, tumour engraftment, and proliferation patterns were analysed every 3 days.

### 2.6 Histopathology and immunophenotype

For histological examination, an autopsy was performed on each passage of nude mice. The samples were fixed in 10% neural‐buffered formalin, embedded in paraffin. Four‐micrometre‐thick sections were made and stained with haematoxylin and eosin. Morphologic observation included architecture of the tumours and distribution of the tumour cells, the size and shapes of the tumour cells, the number of mitotic figures and other combined changes including necrosis, angiocentric invasion and so on. Moreover, we evaluated tumour cells from the mouse models in terms of characteristics of cell surface antigens associated with B cell origin and cell adhesion molecules.

### 2.7 In situ hybridization

Epstein Barr virus (EBV) encoded EBV‐encoded RNA in situ hybridization (RISH) was performed to detect RNA transcripts of EBV using RISH Epstein Barr encoded RNA probe kit (ZSGB‐BIO, Beijing, China, ISH‐6022‐Y, 170510). Tissue sections were deparaffinized, rehydrated, and digested with proteinase K enzyme. After pretreatment in 10 mmol L^−1^ sodium citrate (pH 6.0), sections were hybridized with digoxygenin labelled RISH EBER probe by incubation at 55°C for 1 hour. Following hybridization, sections were washed with tris buffer (pH 7.6) and hybridized probe are detected and diaminobenzidene treatment followed by counterstaining with haematoxylin. Staining was observed under light microscope. Dark brown staining of the tumour nucleus was considered as positive for EBER transcripts expression.

### 2.8 Flow cytometry (FCM) analysis

To evaluate surface antigens associated with cell adhesion and with the cell of origin as the normal counterpart of the tumour cells, FCM analyses were performed using the target antigens including CD2, CD3, CD4, CD5, CD7, CD8, CD45, CD56, CD57, and Ki‐67. All antibodies were obtained from Beckman Coulter (Kraemer Boulevard Brea, CA, USA). Briefly, trypsinized cells, prepared as a single cell suspension, were suspended in 50 μL aliquots of 0.1% bovine serum albumin‐PBS balanced salt solution (BSA‐PBS). Fluorophore‐conjugated CD antibodies were added to the cell suspensions and incubated for 1 hour at room temperature in the dark. Isotype‐specific fluorochromated antibodies were used as negative controls to delineate the negative cell population. The reaction was stopped by the addition of 1 mL of 0.1% BSA‐PBS. The cells were centrifuged and washed twice suspended in PBS. Flow analysis was done by a BD machine (FACS Canto TM, BD, Piscataway, NJ, USA) fitted with proper lasers. Events that registered outside this trace were scored as positive, and 50 000 events were collected for each sample. The percentage of positive events was determined.

### 2.9 Statistical analysis

Two‐tailed *t* tests of equal variance were used to analyse flow cytometry data. Bioluminescence was evaluated using unpaired *t* tests. Statistical significance was defined as a *P* value of less than or equal to 0.05. All statistical analyses were done by using Prism 5.

## 3 RESULT

### 3.1 Time‐lapse engraftment analysis of the NK/T cell lymphoma

We analysed tumour engraftment and proliferation in the mouse models over a time course. The human NK/T cell lymphoma cell lines YT and NKYS cells were injected into one 4‐week‐old female mouse. The 1st passage of the above tumours was visible 40 and 30 days, respectively, later after the injection. Subsequent passages were performed with serial tumour suspension injection when tumours grew up to 1.0‐2.0 cm at length. The mice often showed cachexia in the late stage. Up to now, the two kinds of lymphomas have been passaged to the 7th and 10th generation, the autopsy findings of the mouse models are shown in Figure S1. The combined data suggest that, at least in these models, the tumour cells were not disseminated (data not shown). Each passage of tumour tissue suspension and/or cells was injected into the next mice with matrigel. The pieces of fresh transplanted tumour samples in 7th passage were taken from the mice, and transplanted directly into the right axillary region of 10 mice respectively. The NKYS and YT transplanted tumours had appeared in all of the 9 NOD/SCID mice, but YT in all of 7 nude mice. The growth rate is not different in every passage (data not shown) and the growth curve in the last passage is shown in Figure S1.

### 3.2 Tissue pathologic features

**Figure 1:**
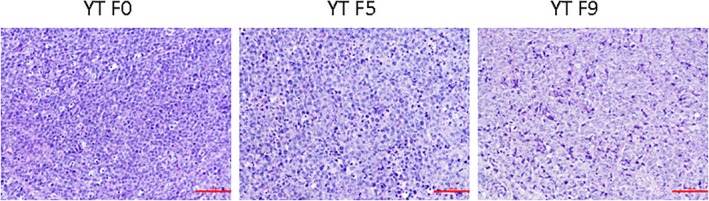
In YT and its serial passage mice models tissues, the normal architecture was effaced, heterogeneous lymphocytes diffused into the plate, with big volume and abundant cytoplasm. The karyotype in cells is irregular and the chromatin is coarse. apoptotic and necrotic tumour cells were found

**Figure 2:**
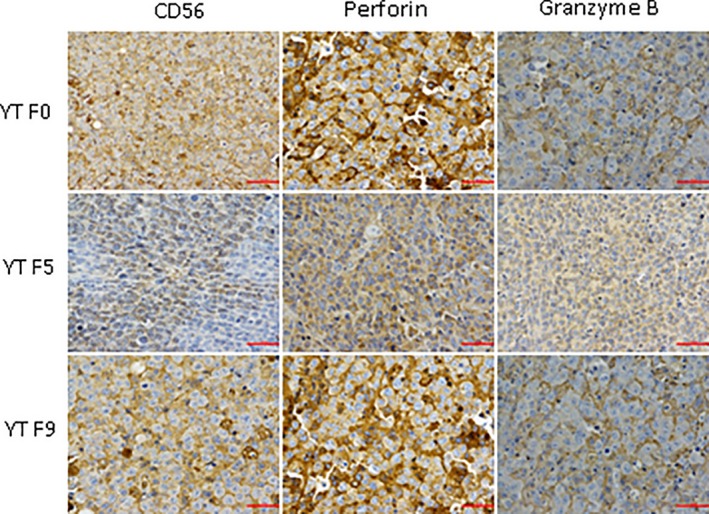
The tumour tissues in YT F0, YT F5, and YT F7 were positive for CD56, Granzyme B, Perforin

The cells in the process of passage had the same feature with the original cell lines. In YT and its serial passage mice models tissues, the normal architecture was effaced, heterogeneous lymphocytes diffused into the plate, with big volume and abundant cytoplasm. The karyotype in cells is irregular and the chromatin is coarse. apoptotic and necrotic tumour cells were found (Figure 1). The tumour tissues in YT F0, YT F5 and YT F7were positive for CD56, Granzyme B, Perforin (Figure 2) but negative for TiA1(Table 1).

**Table 1:** The expression of antigen in tissue of two models

Antigen	YT F0	YT F5	YT F9	NKYS F0	NKYS F4	NKYS F7
CD3	−	−	−	−	−	−
CD4	−	−	−	−	−	−
CD8	−	−	−	−	−	−
CD20	−	−	−	−	−	−
CD56	+	+	+	+	+	+
TiA1	−	−	−	+	+	+
Granzyme B	+	+	+	+	+	+
Perforin	+	+	+	+	+	+
EBER	+	+	+	+	+	+

**Figure 3:**
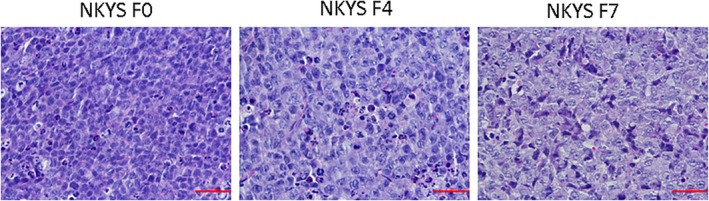
Sections of the biopsy from serial NKYS cell mice models showed similar morphological features with YT mouse model tissues

**Figure 4:**
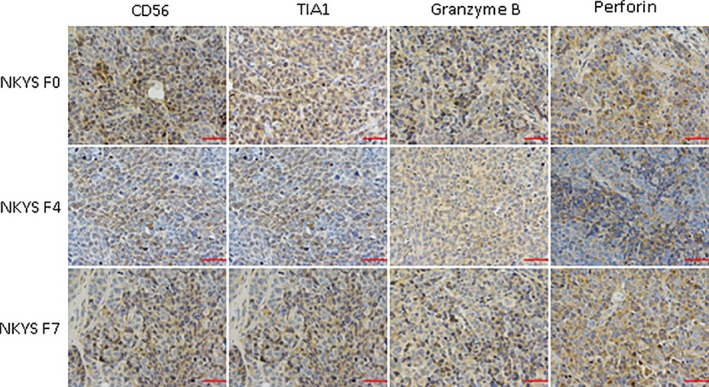
Immunohistochemical staining in NKYS models showed the large atypical cells were positive for CD56, Granzyme B, Perforin, TiA1

Sections of the biopsy from serial NKYS cell mice models showed similar morphological features with YT mouse model tissues (Figure 3). Immunohistochemical staining showed the large atypical cells were positive for CD56, Granzyme B, Perforin, TiA1 (Figure 4 and Table 1).

All characteristics in both kinds of biopsies are consistent with NK/T cell lymphoma. The expression of cell surface markers is summarized in Table 1.

### 3.3 Cell lines features

**Figure 5:**
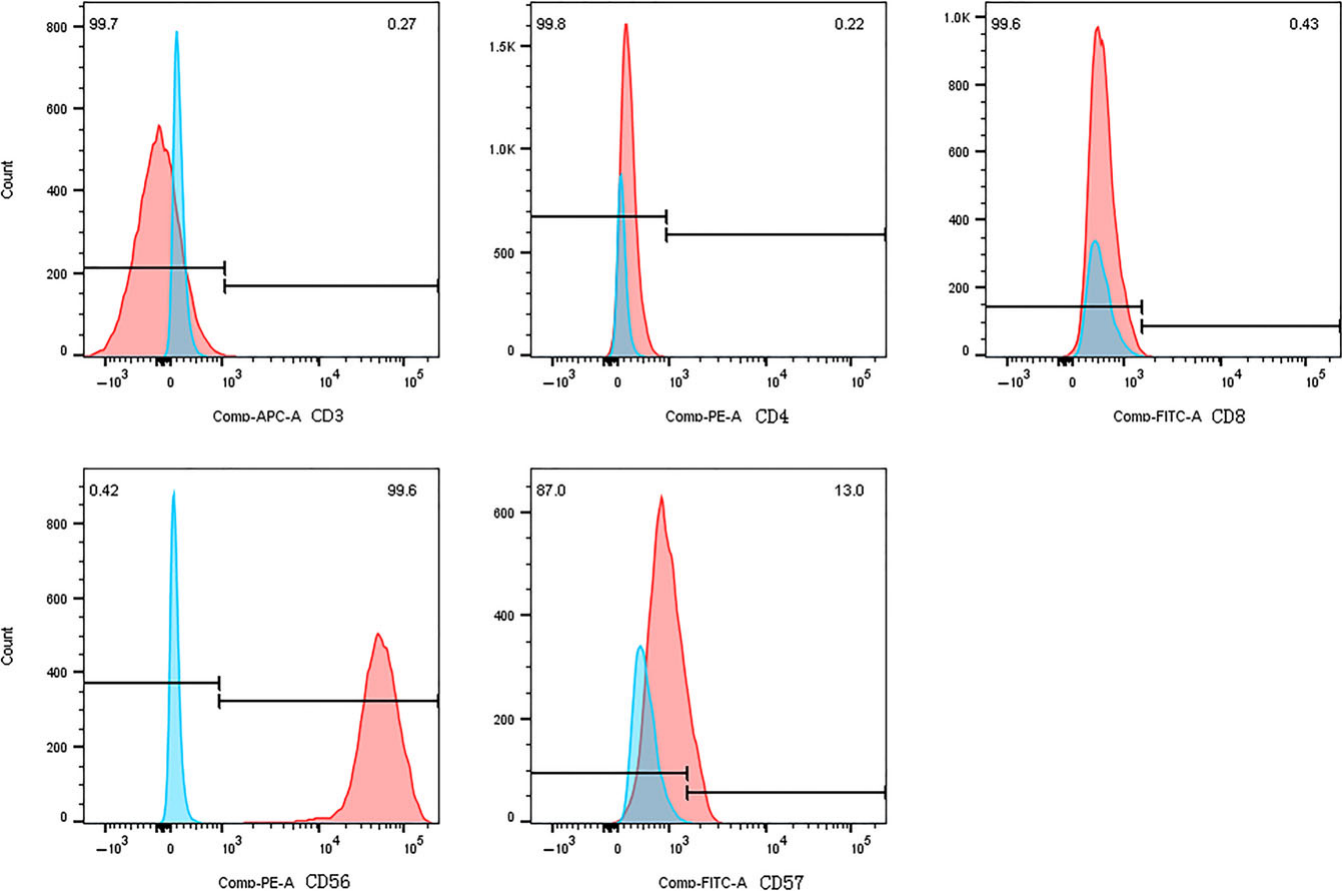
YT F0, YT F5, and YT F9 cells were CD3−/CD4−/CD8−/CD45+/CD56+/CD57−. Blue colour represents unstained samples and red colour represents stained samples

**Figure 6:**
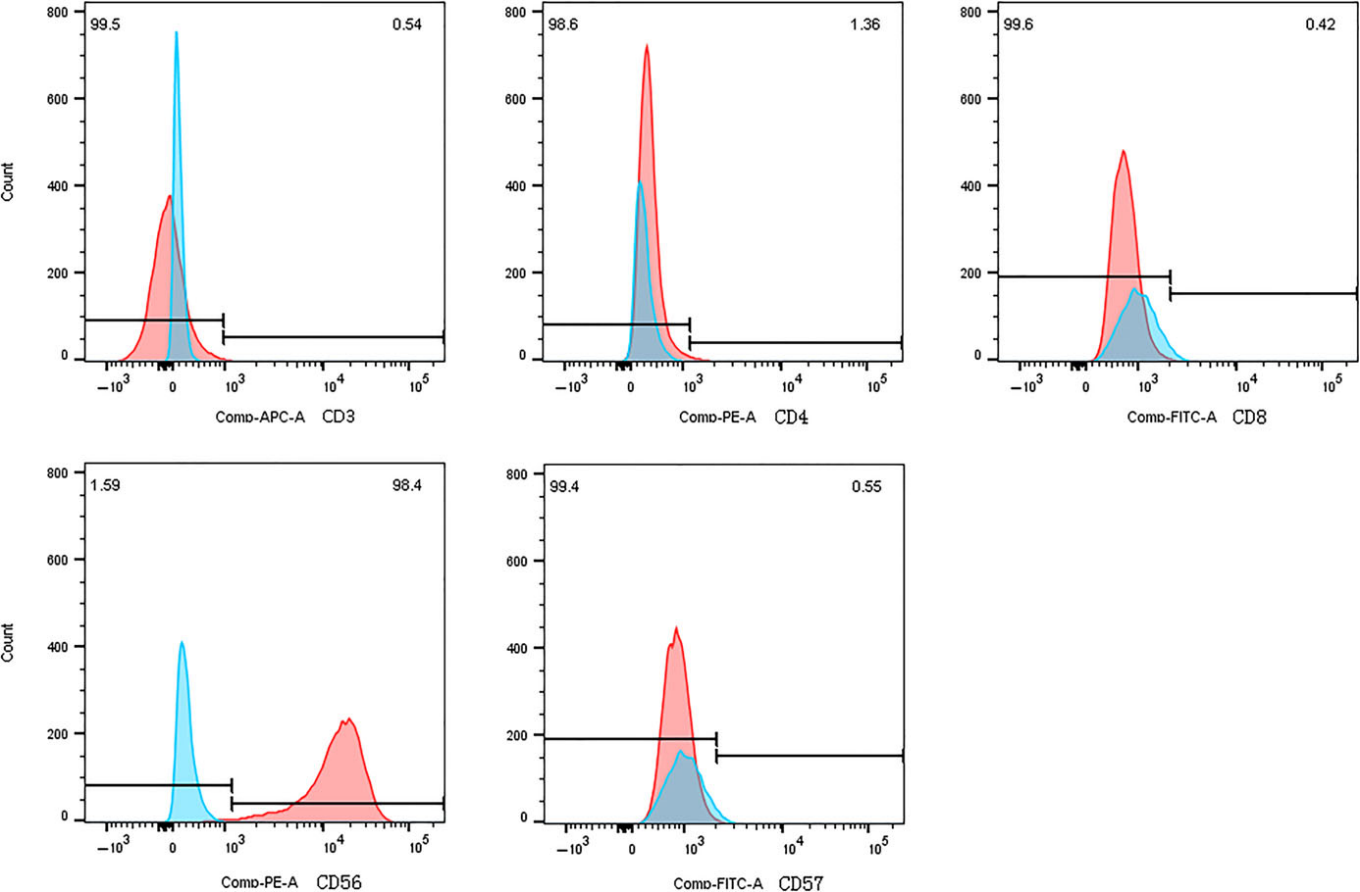
The serial NKYS F0, NKYS F4, and NKYS F7 cells were CD3−/CD4−/CD8−/CD45+/CD56+/CD57−

In the process of passage, the cells of every passage tumour obtained through the strainer were cultured in the same environment with the original cell lines. The cultured cells have continued to proliferate with the same morphology of original cell lines. Cytocentrifuge smears of the cells were prepared and stained with May‐Giemsa for morphologic evaluation. The cells can be cryopreserved in cryopreservation‐medium (90% FBS, 10% DMSO), stored in liquid nitrogen, thawed again (with a viability of more than 70%) and successfully reconstituted. Flow cytometry analysis was performed on cell suspensions cultured from each passage tumour tissues. YT F0, YT F5, YT F9 cells were CD3−/CD4−/CD8−/CD45+/CD56+/CD57− (Figure 5 and Table 2). Similar to YT cells, the serial NKYS F0, NKYS F4, NKYS F7 cells were CD3−/CD4−/CD7+/CD8−/CD45+/CD56+/CD57−. No difference existed between passages (Figure 6 and Table 2).

**Table 2:** The detection of antigen in flow

	YT F0	YT F5	YT F9	NKYS F0	NKYS F4	NKYS F7
CD3	−	−	−	−	−	−
CD4	−	−	−	−	−	−
CD8	+	+	+	+	+	+
CD38	+	+	+	+	+	+
CD45	+	+	+	+	+	+
CD56	+	+	+	+	+	+
CD57	−	−	−	−	−	−

### 3.4 EBV infection

**Figure 7:**
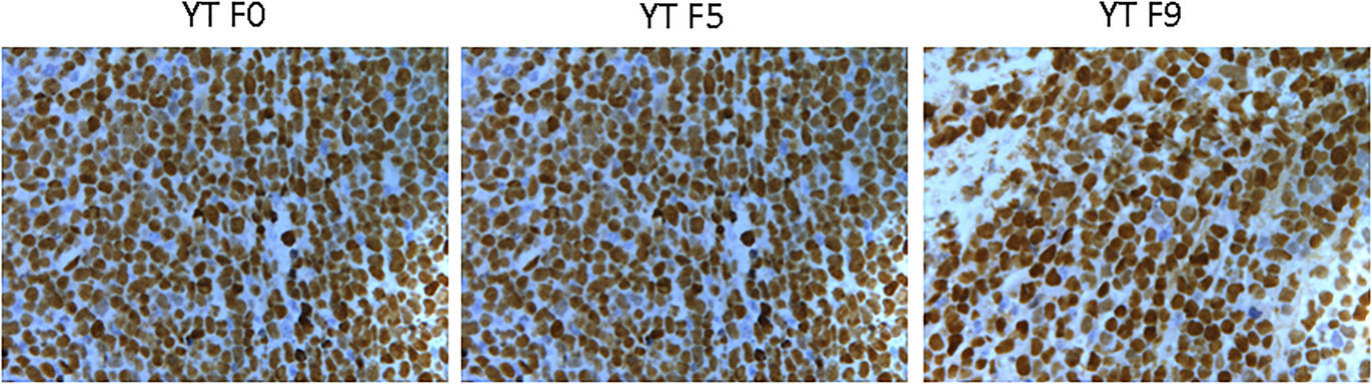
EBV was strongly detected in situ hybridization for EBV RNA using the EBER probe in YT and later passage tissues

**Figure 8:**
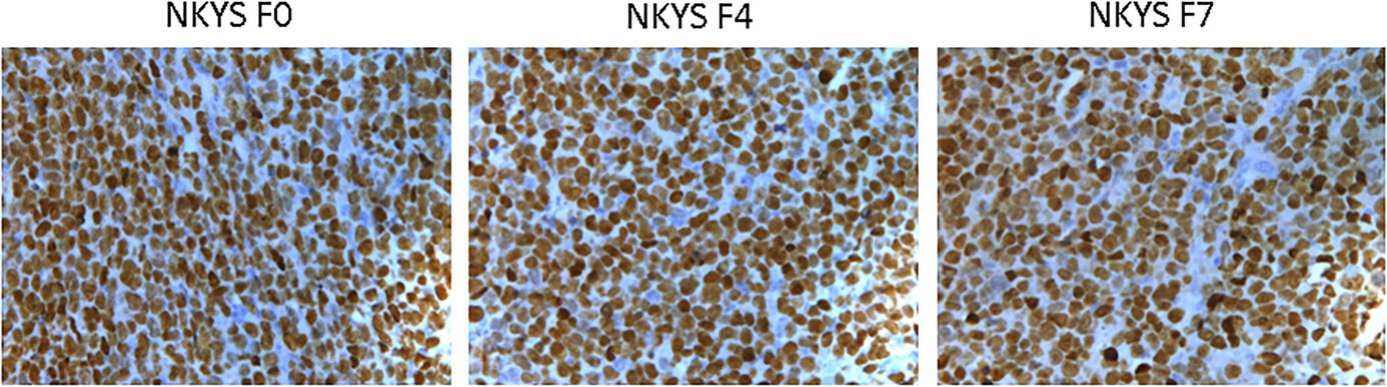
EBV was strongly detected in situ hybridization for EBV RNA using the EBER probe in NKYS and later passage tissues

EBV was strongly detected in situ hybridization for EBV RNA using the EBER probe in YT, NKYS, and later passage tissues (Figures 7 and 8; Table 1).

## 4 DISCUSSION

In vitro studies have always been a powerful tool to characterize many cancer‐associated processes. However, the tumours comprise not only the malignant cells but also a plethora of nonmalignant cells and extracellular matrix‐the tumour microenvironment,^11^ that's why numerous aspects of neoplastic growth are difficult to model in vitro alone. In vitro approach has limitations when we extrapolate to the more complex in vivo scenario.^12^ In vivo strategies are expected to incorporate tumour micro environmental clues that cannot be easily modelled in vitro. The latter applies to mechanisms of resistance to therapeutic, including tumour cell intrinsic mechanisms of resistance to low dose metronomic chemotherapy.^13^, ^14^ While in vivo passage is an increasingly applied strategy to improve the modelling of therapeutic resistance.^15^, ^16^ Severe immunodeficient mice (SCID) mice have widely been used to disseminate tumour cells in vivo,^17^ where the cells are engrafted via intravenous injection.^18^, ^19^ These mice have been used to develop a mouse model for human Burkitt lymphoma by using the Daudi cell line or SU‐DHL‐4 cells.^20^ Improvements in mouse technology have facilitated research and preclinical studies in lymphoma fields, especially the application of NOD.Cg‐*Prkdc* ^*scid*^ *Il2rg* ^*tm1Wjl*^/SzJ (NSG) mice in Mice engrafted with human tumours act as a model for testing various therapeutic drugs for their potency, toxicity, and dosing.^21^ Actually, The development of NSG mice has provided a valuable tool for the development of a NK/T cell lymphoma model, because they lack mature B and T cells and various cytokines such as IL2, 4, 7, 9, 15, and 21, leading to impaired development of NK cells.^22^, ^23^ However, stable passages in lymphoma models have not been reported. It is admitted that NK/T cell lymphoma research has less success and popular in vivo. On one hand, NK/T cell lymphoma mouse models are hard to be established. The tumours are hard to grow after cells injection because of the stimulation of immune environment in vivo and extra vivo. On the other hand, even if the models are established successfully, they remain not stable in passages, or never have been passaged. So far, only some Japanese established SNK6‐which is a NK/T cell lymphoma cell line‐mouse model in NOG mice, which is a similar species to NSG.^24^, ^25^ It is known that using NSG and NOG mice to establish models costs too much, meanwhile because of the patent problem, many enthusiastic researchers have not the opportunity to use them. Therefore, if we could find one economic and efficient way to attain the same aim, that will benefit more researchers in the world. Fortunately, we have filled the historical blank with NK/T cell lymphoma mouse models, and in vivo passage makes it possible to screen and evaluate the novel antitumour drugs in NK/T cell lymphoma.

It was reported that there is some discrepancy in different passage of mouse models, for instance, Metildi et al^26^ ever reported that serially passaging in vivo generated more aggressive variants of both human pancreatic cancer cell lines, one of which was Kras wild‐type (BXPC‐3) and the other Kras mutant, Panc‐1, which displayed faster tumour growth and shortened survival time. To define the characteristics of tumours after passage, we examine the cell surface markers in IHC and Flow cytometry, EBV in ISH. YT cells, known as natural killer (NK) cell lines, resulted from a patient with acute lymphoblastic lymphoma with thymoma.^27^ NKYS cells preserved the original characteristics of EBV‐associated nasal angiocentric T/NK cell lymphoma.^28^ These two cell lines are successfully representative of the typical NK/T cell lymphoma occurring in the world. In our mouse models, there is no significant difference in growth rate, histopathological, and immunohistochemical features among different passages of YT and NKYS models, which largely proves that we have got stable mouse models. More importantly, we invent a method to establish the NK/T mice model. CTX is necessary before injecting and implanting so that the immune system of mice can be inhibited more thoroughly. However, NKYS model is still hard to establish. In terms that NKYS cell line is dependent on IL‐2, We hypnosis that the tumour need IL‐2 to grow. It is critical that the tumour itself need the same environment with the corresponding cell line. In the future, the mouse models will facilitate the preclinical research of NK/T cell lymphoma.

However, using cell lines as inoculum an IL‐2 support does barely reflect “real world” inter‐patient heterogeneity of the lymphomas because the subcutaneously injected cells did not disseminate beyond the injection site. That is a world‐wild question and there is a long way to go in the research on establishing mouse models that can mimic the real tumour environment in patients.

## References

[1] Tse E , Kwong YL . The diagnosis and management of NK/T‐cell lymphomas. J Hematol Oncol. 2017;10:85 10.1186/s13045-017-0452-9. 28410601 PMC5391564

[2] Suzuki R , Suzumiya J , Yamaguchi M , et al. Prognostic factors for mature natural killer (NK) cell neoplasms: aggressive NK cell leukemia and extranodal NK cell lymphoma, nasal type. Ann Oncol. 2010;21:1032‐1040. 10.1093/annonc/mdp418. 19850638

[3] Chim CS , Ma SY , Au WY , et al. Primary nasal natural killer cell lymphoma: long‐term treatment outcome and relationship with the International Prognostic Index. Blood. 2004;103:216‐221. 10.1182/blood-2003-05-1401. 12933580

[4] Kwong YL , Kim WS , Lim ST , et al. SMILE for natural killer/T‐cell lymphoma: analysis of safety and efficacy from the Asia Lymphoma Study Group. Blood. 2012;120:2973‐2980. 10.1182/blood-2012-05-431460. 22919026

[5] Lee J , Suh C , Park YH , et al. Extranodal natural killer T‐cell lymphoma, nasal‐type: a prognostic model from a retrospective multicenter study. J Clin Oncol. 2006;24:612‐618. 10.1200/JCO.2005.04.1384. 16380410

[6] Au WY , Weisenburger DD , Intragumtornchai T , et al. Clinical differences between nasal and extranasal natural killer/T‐cell lymphoma: a study of 136 cases from the International Peripheral T‐Cell Lymphoma Project. Blood. 2009;113:3931‐3937. 10.1182/blood-2008-10-185256. 19029440

[7] Wu L , Li Y , Fan JM , et al. MicroRNA‐204 targets signal transducer and activator of transcription 5 expression and inhibits proliferation of B‐cell lymphoma cells. Mol Med Rep. 2015;11:4567‐4572. 10.3892/mmr.2015.3298 25651400

[8] Ponce RA , Gelzleichter T , Haggerty HG , et al. Immunomodulation and lymphoma in humans. J Immunotoxicol. 2014;11:1‐12. 10.3109/1547691X.2013.798388. 23746314

[9] Bernardi R , Grisendi S , Pandolfi PP . Modelling haematopoietic malignancies in the mouse and therapeutical implications. Oncogene. 2002;21:3445‐3458. 10.1038/sj.onc.1205313. 12032781

[10] Zhang Y , Nagata H , Ikeuchi T , et al. Common cytological and cytogenetic features of Epstein‐Barr virus (EBV)‐positive natural killer (NK) cells and cell lines derived from patients with nasal T/NK‐cell lymphomas, chronic active EBV infection and hydroa vacciniforme‐like eruptions. Br J Haematol. 2003;121:805‐814. 12780797 10.1046/j.1365-2141.2003.04359.x

[11] Hanahan D , Weinberg RA . Hallmarks of cancer: the next generation. Cell. 2011;144:646‐674. 10.1016/j.cell.2011.02.013. 21376230

[12] Cunderlikova B . Issues to be considered when studying cancer in vitro. Crit Rev Oncol Hematol. 2013;85:95‐111. 10.1016/j.critrevonc.2012.06.007. 22823950

[13] Ebos JM , Lee CR , Kerbel RS . Tumor and host‐mediated pathways of resistance and disease progression in response to antiangiogenic therapy. Clin Cancer Res. 2009;15:5020‐5025. 10.1158/1078-0432.CCR-09-0095. 19671869 PMC2743513

[14] Emmenegger U , Francia G , Chow A , et al. Tumors that acquire resistance to low‐dose metronomic cyclophosphamide retain sensitivity to maximum tolerated dose cyclophosphamide. Neoplasia. 2011;13:40‐48. 21245939 10.1593/neo.101174 PMC3022427

[15] Francia G , Cruz‐Munoz W , Man S , Xu P , Kerbel RS . Mouse models of advanced spontaneous metastasis for experimental therapeutics. Nat Rev Cancer. 2011;11:135‐141. 10.1038/nrc3001. 21258397 PMC4540342

[16] du Manoir JM , Francia G , Man S , et al. Strategies for delaying or treating in vivo acquired resistance to trastuzumab in human breast cancer xenografts. Clin Cancer Res. 2006;12:904‐916. 10.1158/1078-0432.CCR-05-1109. 16467105

[17] Kawata A , Yoshida M , Okazaki M , Yokota S , Barcos M , Seon BK . Establishment of new SCID and nude mouse models of human B leukemia/lymphoma and effective therapy of the tumors with immunotoxin and monoclonal antibody: marked difference between the SCID and nude mouse models in the antitumor efficacy of monoclonal antibody. Can Res. 1994;54:2688‐2694. 8168098

[18] Yoshida M , Rybak RJ , Choi Y , et al. Development of a severe combined immunodeficiency (SCID) mouse model consisting of highly disseminated human B‐cell leukemia/lymphoma, cure of the tumors by systemic administration of immunotoxin, and development/application of a clonotypespecific polymerase chain reaction‐based assay. Can Res. 1997;57:678‐685. 9044845

[19] Ghetie MA , Richardson J , Tucker T , Jones D , Uhr JW , Vitetta ES . Disseminated or localized growth of a human Bcell tumor (Daudi) in SCID mice. Int J Cancer. 1990;45:481‐485. 2307538 10.1002/ijc.2910450318

[20] Yan JS , Chen XY , Li WP , Yang Y , Song ZL . Establishing SCID mouse models of B‐cell non‐Hodgkin's lymphoma. Chinese J Cancer. 2009;28:181‐183. 19550134

[21] Brehm MA , Shultz LD , Greiner DL . Humanized mouse models to study human diseases. Curr Opin Endocrinol Diabetes Obes. 2010;17:120‐125. 10.1097/MED.0b013e328337282f. 20150806 PMC2892284

[22] Leonard JE , Johnson DE , Felsen RB , Tanney LE , Royston I , Dillman RO . Establishment of a human B‐cell tumor in athymic mice. Can Res. 1987;47:2899‐2902. 3105870

[23] Shultz LD , Ishikawa F , Greiner DL . Humanized mice in translational biomedical research. Nat Rev Immunol. 2007;7:118‐130. 10.1038/nri2017. 17259968

[24] Yamada D , Iyoda T , Vizcardo R , et al. Efficient regeneration of human Valpha24(+) invariant natural killer T cells and their anti‐tumor activity in vivo. Stem Cells. 2016;34:2852‐2860. 10.1002/stem.2465. 27422351

[25] Kawada J , Ito Y , Iwata S , et al. mTOR inhibitors induce cell‐cycle arrest and inhibit tumor growth in Epstein‐Barr virus‐associated T and natural killer cell lymphoma cells. Clin Cancer Res. 2014;20:5412‐5422. 10.1158/1078-0432.CCR-13-3172. 25208880

[26] Metildi CA , Kaushal S , Hoffman RM , Bouvet M . In vivo serial selection of human pancreatic cancer cells in orthotopic mouse models produces high metastatic variants irrespective of Kras status. J Surg Res. 2013;184:290‐298. 10.1016/j.jss.2013.03.049. 23590868 PMC3724759

[27] Tani A , Tatsumi E , Nakamura F , Kumagai S . [Epstein‐Barr virus (EBV)‐positive NK cell line YT, and ALL/LBL of NK‐lineage]. Rinsho Byori. 1996;44:936‐943. 8937183

[28] Uno M , Tsuchiyama J , Moriwaki A , et al. In vitro induction of apoptosis for nasal angiocentric natural killer cell lymphoma‐derived cell line, NK‐YS, by etoposide and cyclosporine A. Br J Haematol. 2001;113:1009‐1014. 11442496 10.1046/j.1365-2141.2001.02844.x

