# Corrigendum

**DOI:** 10.1111/jcmm.17775

**Published:** 2023-06-16

In Xue W et al.,^1^ Figures 2, 4 and 7 were incorrect due to an error in the preparation of these figures for publication. It was noticed that the image for CD56 and Perforin of YT F9 in Figure 2, were coming from photographs of YT F0. In addition, the images for TIA1 of NKYS F4 and F7 in Figure 4, the image for EBER of YT F5 in Figure 7, are the same with the left panel (images for CD56 of NKYS F4 and F7 in Figure 4, the image for EBER of YT F0 in Figure 7). The corrected figures appear below. The authors confirmed all results and conclusions of this article remain unchanged. We apologize for any confusion this may have caused.

**FIGURE 2:**
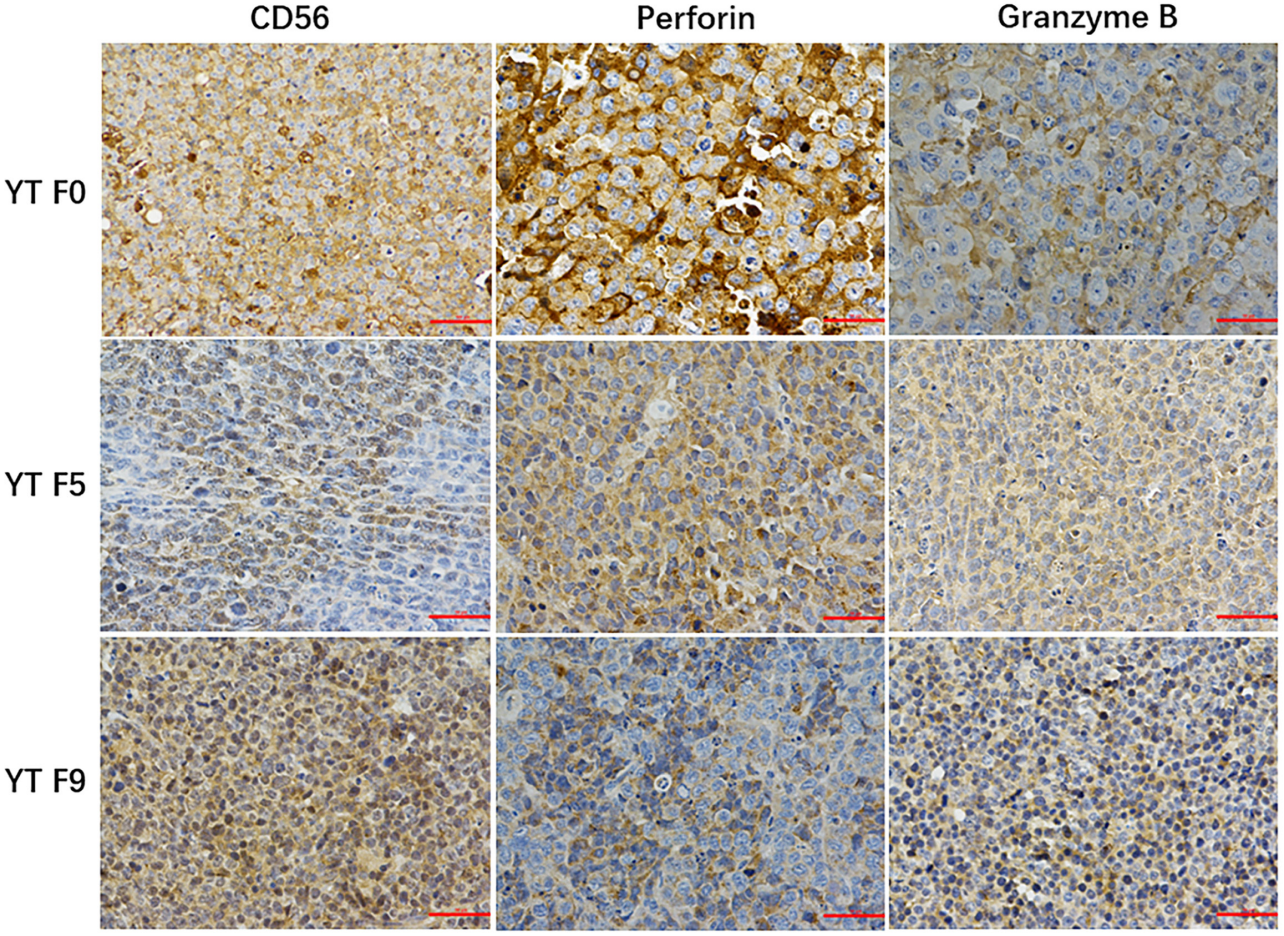
The tumour tissues in YT F0, YT F5, and YT F7 were positive for CD56, Granzyme B, Perforin.

**FIGURE 4:**
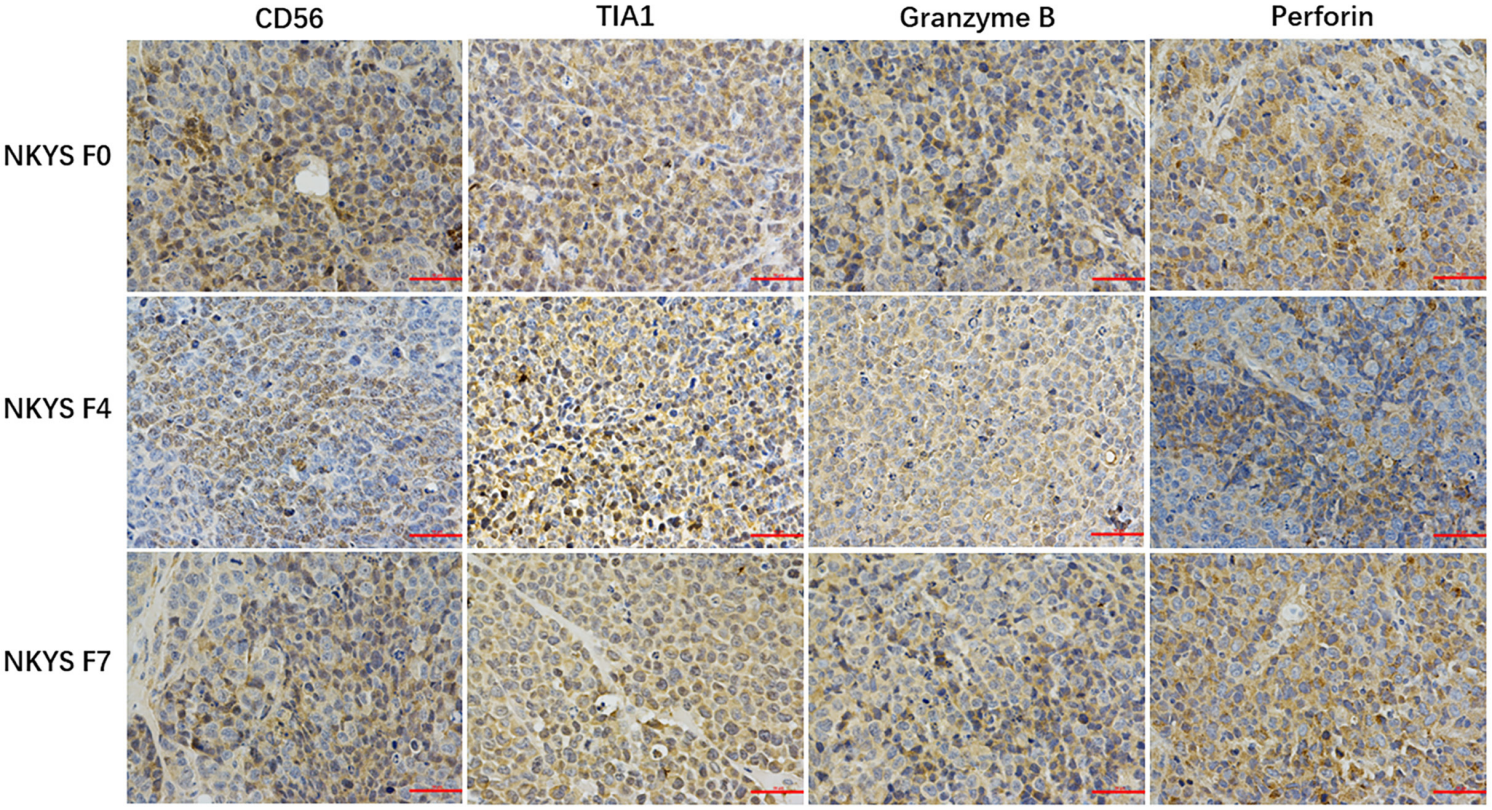
Immunohistochemical staining in NKYS models showed the large atypical cells were positive for CD56, Granzyme B, Perforin, TiA1.

**FIGURE 7:**
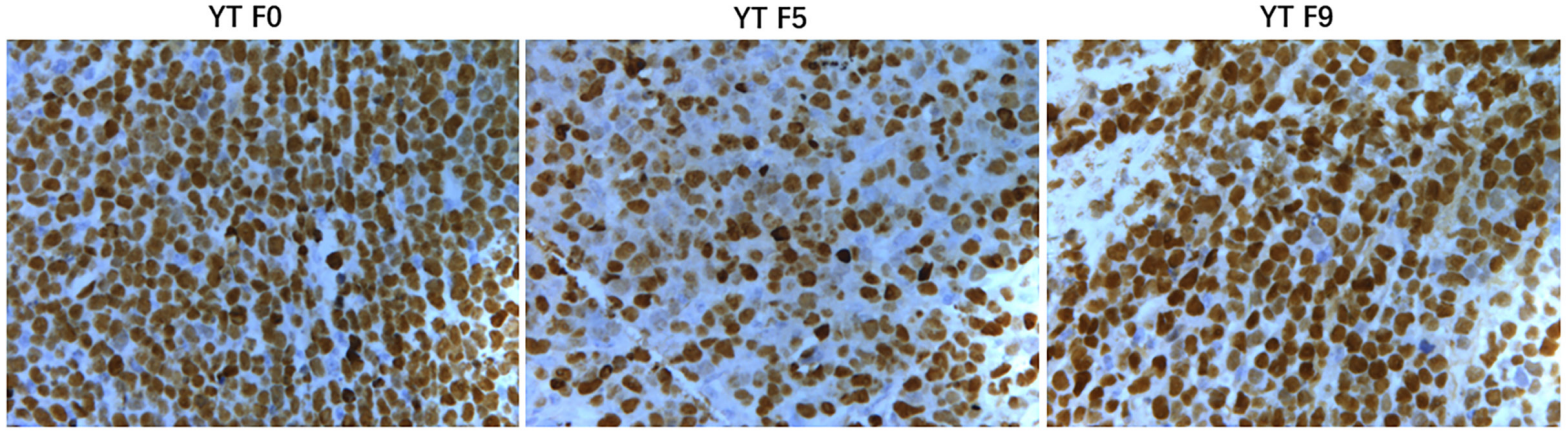
EBV was strongly detected in situ hybridization for EBV RNA using the EBER probe in YT and later passage tissues.
