# Supplementary figures and images for: One method to establish Epstein‐Barr virus‐associated NK/T cell lymphoma mouse models
Source: J Cell Mol Med. 2018 Nov 28;23(2):1509–16. doi: 10.1111/jcmm.14057 (PMC6349153; doi:10.1111/jcmm.14057)

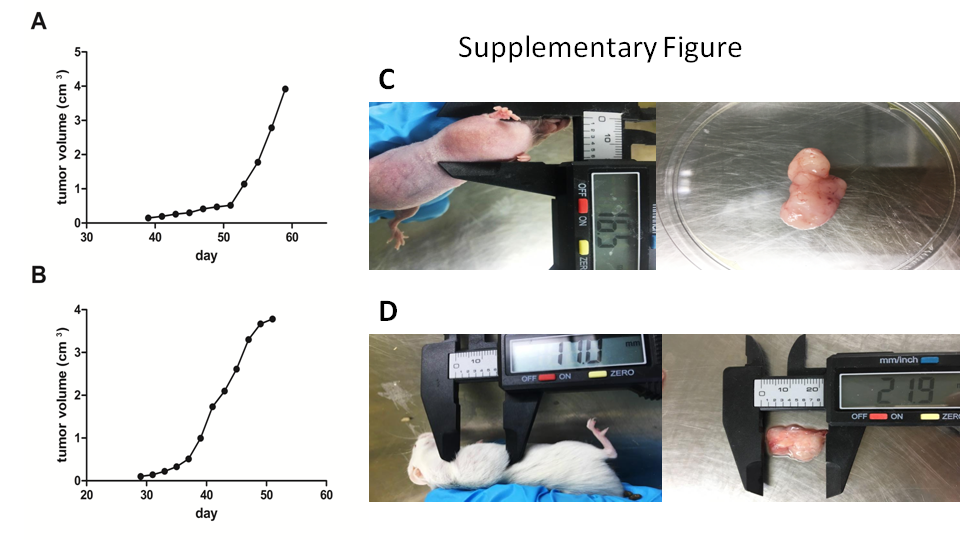

Supplement: Supplementary file 1 [file JCMM-23-1509-s001.TIF]
